# Transcript annotation tool (TransAT): an R package for retrieving annotations for transcript-specific genetic variants

**DOI:** 10.1186/s12859-021-04243-z

**Published:** 2021-06-28

**Authors:** Ching-Yu Shih, Amrita Chattopadhyay, Chien-Hui Wu, Yu-Wen Tien, Tzu-Pin Lu

**Affiliations:** 1 Bioinformatics and Biostatistics Core, Centre of Genomic and Precision Medicine, National Taiwan University, Taipei, 10055 Taiwan; 2 Department of Public Health, Institute of Epidemiology and Preventive Medicine, National Taiwan University, Taipei, 10055 Taiwan; 3 Department of Surgery, National Taiwan University Hospital, Taipei, Taiwan

**Keywords:** Transcript annotation, Variant annotation, R package, TransAT, Allele frequency

## Abstract

**Background:**

An individual’s genetics play a role in how RNA transcripts are generated from DNA and consequently in their translation into protein. Transcriptional and translational profiling of patients furnishes the information that a specific marker is present; however, it fails to provide evidence whether the marker correlates with response to a therapeutic agent. A comparative analysis of the frequency of genetic variants, such as single nucleotide polymorphisms (SNPs), in diseased and general populations can identify pathogenic variants in individual patients. This is in part because SNPs have considerable effects on protein function and gene expression when they occur in coding regions and regulatory sequences, respectively. Therefore, a tool that can help users to obtain the allele frequency for a corresponding transcript is the need of the day. Several annotation tools such as SNPnexus and VariED are publicly available; however, none of them can use transcript IDs as input and provide the corresponding genomic positions of variants.

**Results:**

In this study, we developed an R package, called transcript annotation tool (TransAT), that provides (i) SNP ID and genomic position for a user-provided transcript ID from patients, and (ii) allele frequencies for the SNPs from publicly available global populations. All data elements are extracted, collected, and displayed in an easily downloadable format in two simple command lines. TransAT is available on Windows/Linux/MacOS and is operative for R version 4.0.4 or later. It is available at https://github.com/ShihChingYu/TransAT and can be downloaded and installed using devtools::install_github("ShihChingYu/TransAT", force=T) on the R execution page. Thereafter, all functions can be executed by loading the package into R with library(TransAT).

**Conclusions:**

TransAT is a novel tool that seamlessly provides genetic annotations for queried transcripts. Such easily obtainable information would be greatly advantageous for physicians, assisting them to make individualized decisions about specific drug treatments. Moreover, allele frequencies from user-chosen global ethnic populations will highlight the importance of ethnicity and its effect on patient pathogenicity.

**Supplementary Information:**

The online version contains supplementary material available at 10.1186/s12859-021-04243-z.

## Background

Transcriptome profiling can potentially reveal the molecular precursors of disease in ethnically diverse samples, long before the disease symptoms become evident. Gene expression profiling and metabolite profiling are two methods that are typically used to uncover molecular processes and candidate genes that underlie the prevalence and outcome of conditions such as cardiovascular, metabolic, neurodegenerative diseases [1] and cancer [2, 3]. A messenger RNA (mRNA) transcript is generated from DNA and is consequently translated into protein [4]. Genetic variants, such as single nucleotide polymorphisms (SNPs), have been characterized in terms of their co-dominance, reproducibility, locus-specificity, and random genome-wide distribution [5], and a detailed analysis of SNPs can identify pathogenic ones in individual patients. This is in part because SNPs have considerable effects on protein function when they occur in coding sequences and on gene expression when they occur in regulatory regions [6]. Hence, SNPs are the ideal candidates for genetic research, leading to functional characterization and identification of associated traits.

Accurate annotations of SNPs, including gene sequences, amino acid changes, metabolic impacts, associated diseases, and population frequencies, from different ethnicities and disease populations allows better biological interpretation. This further helps in the discovery of novel putative genetic factors through disease association studies and differential mutation analyses, towards uncovering underlying disease mechanisms [7]. Also, the variant allele frequencies provide an understanding of the distribution of disease-associated variants in reference populations [8]. Several SNP annotation tools are already available for easy public access. SNPnexus, a popular tool that provides detailed annotation of coding and noncoding variants, can identify potential therapeutic targets; however, it requires the genomic position, and chromosomes or contigs as inputs, in order to characterize outcomes of the transcriptomes and the proteomes. It maps variants to publicly available human variation catalogs and obtains genotype data and corresponding allele frequencies [9]. Similarly, VariED, an annotation tool developed by our research group, works by accepting genomic positions and allele information as input, thereby providing the users with annotations, functional and clinical consequences of the corresponding queried SNP [10]. As yet, there is no tool available that can accept transcript IDs as input and provide the corresponding genomic positions of variants. Obtaining genomic positions of SNPs within the transcriptome is possible, but it involves a series of data-mining steps using several existing online databases and is a labor-intensive process when done manually. The steps involve pre-processing (to correct the search format), searching related information based on RefSeq ID / UCSC ID, and mapping the positions of transcripts to the genome. These steps can be strung together in custom ways, but a tool able to perform all steps in a single environment would make such analyses accessible to a wider range of scientists and clinicians. The R programming language enables powerful analysis and visualization of complex data and is widely used by the biomedical research community. Even though there exist several R packages that can conduct certain steps of the overall pipeline, there are few that can execute the entire pipeline in a comprehensive and reproducible way. Thus, a transcript annotation package within R would be beneficial for bioinformatics non-experts, especially physicians.

Therefore, in this study, for easy access to individual genetic information, we have developed an R package, called transcript annotation tool (TransAT), that provides (i) SNP ID and genomic position for a user-provided transcript ID from patients, and (ii) allele frequencies for the SNPs from publicly available global populations. All outputs are curated and displayed in an easy to obtain downloadable format. Users are required to use two simple command lines that conduct preprocessing, data transformation, data extraction, and data visualization automatically, to provide users with all the desired information.

Transcriptional and translational profiling of patients furnishes the information that a specific marker is present; however, it fails to provide evidence whether the marker correlates with response to a therapeutic agent [11]. Allele frequency of a variant in the general population is an important reference point for conducting disease studies. It provides evidence whether a SNP may be potentially pathogenic in a disease population, based on the degree of its deviance from the reference population [12, 13]. Therefore, a tool that can help users to obtain allele frequency for a corresponding transcript is the need of the day. TransAT can easily provide genetic annotations and allele frequencies for queried transcripts, thereby providing clinicians easy access to information that may help them with treatment decisions. Moreover, allele frequencies from diverse populations will highlight the effect of ethnicity, if any, on patient pathogenicity [14].

## Implementation

### Overview of TransAT workflow

**Fig. 1:**
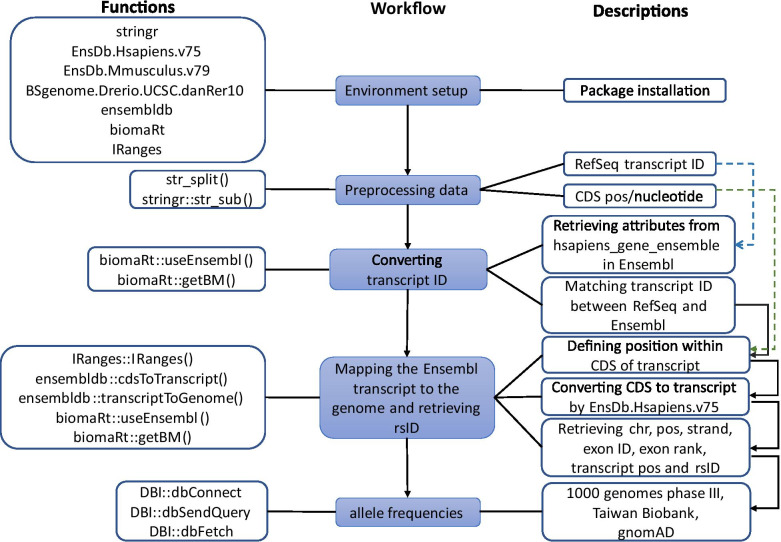
Overview of the workflow of Transcript Annotation Tool (TransAT). (Center) Workflow: displays each working step for TransAT, sequentially. (Left) Functions: provides the list of functions used in the backend programming for conducting each step of the workflow. (Right) Descriptions: provides simple descriptions to explain each step of the workflow. CDS: coding sequence

TransAT was developed as a package in the R programming language for converting RefSeq ID / UCSC ID [15, 16] to Ensembl transcripts [17] and mapping the genomic position, followed by assigning variant annotations. An overview of TransAT is illustrated in Fig. 1. The purpose was to provide a simplified way to conduct a series of conversions and query variant annotations, implemented through a standardized workflow, providing reproducible, easily interpretable outputs in simplified formats. TransAT is available on Windows, Linux, and macOS operating systems and can be used in an R-interactive version or in the background. The package is operative for R version 4.0.4 or later. All source code is freely available at GitHub (https://github.com/ShihChingYu/TransAT) and all relevant information is provided in Additional File 1:

### Package contents

TransAT primarily contains information for transcriptomes from humans (though access to zebrafish and mouse genomes has recently been added; see below), and provides amino acid changes, gene names, and corresponding genomic (variant) positions and annotations for the human genome. It accesses allele frequencies for different global populations from the publicly available 1000 Genomes [18], The Genome Aggregation Database (gnomAD) [19, 20] and Taiwan Biobank (TWB) [21] databases. The 1000 Genomes phase 3 data is constructed of East Asians, Americans, Africans, Europeans, and South Asians. GnomAD contains variants from 123,136 exomes and 15,496 genomes from different ethnic populations such as Latino, African, Ashkenazi Jewish, European, South Asian, and East Asian. TWB constitutes of SNP array data from 21,695 Taiwanese individuals and next-generation sequencing SNP data from 1,517 Taiwanese individuals.

### Package construction and functions

The workflow in TransAT (Fig. 1) starts with a list of RefSeq or UCSC transcripts as input, followed by the getBM function in the biomaRt R package [22]. This step successfully reports the genomic position of the corresponding Ensembl transcript ID. The genomic positions are then used to retrieve rsID and population allele frequencies for further analysis. Two functions, “convert_transcriptID” and “pop_freq”, conduct all the steps, and output intermediate genomic information and final allelic annotation, respectively, based on the user’s requirements.

### Function convert_transcript_id

The input for the convert_transcriptID function consists of a.csv table (Table 1a), where the columns include NCBI RefSeq ID [15], single-nucleotide changes in DNA, and corresponding coding sequence (CDS) position. Each row depicts the transcript information for each individual. TransAT also accepts .BED format as input (Additional File 2: Table S1). The pre-processing step extracts relevant information from user-provided data and feeds it appropriately into each of the subsequent intermediate steps. The reference gene database for humans (hsapiens_gene_ensembl) from the Ensembl database [17] is utilized, and the transcript ID from the NCBI RefSeq database is matched with that from Ensembl to obtain the CDSs based on the nucleotide location and Ensembl transcript, through a function called IRanges (defines classes for storing, transforming, and aggregating large amounts of grouped data). If required, the user can extract any annotation feature from a list of 3,061 options (Additional File 3: Table S2), by specifying it in the R function. The default output parameters consist of chromosome, Ensembl-transcript-id, HGNC-symbol, genomic position (start, end), strand, exon-id, exon-rank, and transcript-position (start, end) and refsnp_id (Table 2a). The CDS position is then mapped to the transcript coordinates utilizing the EnsDb.Hsapiens.v75 package, which connects to the Ensembl human genome reference (hg37) by matching the first nucleotide of the transcript to the genome. This step reports the genomic positions of the variants (SNPs) and the corresponding rsIDs that are obtained from the reference SNP database for humans (hsapiens_snp), for each transcript queried by the user. Command 1 displays the command line required to convert the transcript ID to a variant ID (which yields genomic positions and allele information).

**Table 1:** Input.csv file for the TransAT package

(a)	
Transcript_version	Nucleotide_changes
NM_005359	A947G
NM_000059	C3109T
NM_000059	A4599C

(b)				
Chr	Start	End	Ref	Alt
18	48,586,278	48,586,278	A	G
13	32,911,601	32,911,601	C	T
13	32,913,091	32,913,091	A	C

The input format into the TransAT package for (a) Function: convert_transcriptID(), which converts transcripts to variants and maps them to genomic positions, and (b) Function: pop_freq(), which provides allele frequencies from global populations and gene-based annotations

**Table 2:** Output tables for the TransAT package

(a)																
Transcript_version	Nucleotide_changes	CDS_start_loc	Ref	Alt	Chr	ensembl_transcript_id	hgnc_symbol	start	end	width	strand	exon_id	exon_rank	tx_start	tx_end	refsnp_id
NM_005359	A947G	947	A	G	18	ENST00000342988	SMAD4	48,586,278	48,586,278	1	+	ENSE00003494728	8	1485	1485	rs377119288
NM_000059	C3109T	3109	C	T	13	ENST00000544455	BRCA2	32,911,601	32,911,601	1	+	ENSE00000939168	11	3336	3336	rs80358557
NM_000059	A4599C	4599	A	C	13	ENST00000544455	BRCA2	32,913,091	32,913,091	1	+	ENSE00000939168	11	4826	4826	rs80358694

(b)												
Chr	Start	End	Ref	Alt	gnomAD_exome _ALL_Ref_freq	gnomAD_exome _ALL_Alt_freq	gnomAD_exome _AFR_Ref_freq	gnomAD_exome _AFR_Alt_freq	gnomAD_exome _AMR_Ref_freq	gnomAD_exome _AMR_Alt_freq	gnomAD_exome _ASJ_Ref_freq	gnomAD_exome _ASJ_Alt_freq
18	48,586,278	48,586,278	A	G	0.9999	1.00E−04	1	0	1	0	1	0
13	32,911,601	32,911,601	C	T	1	0	1	0	1	0	1	0
13	32,913,091	32,913,091	A	C	0.9999	1.00E−04	1	0	1	0	1	0

gnomAD_exome_EAS_Ref_freq	gnomAD_exome_EAS_Alt_freq	gnomAD_exome_FIN_Ref_freq	gnomAD_exome_FIN_Alt_freq	gnomAD_exome_NFE_Ref_freq	gnomAD_exome_NFE_Alt_freq	gnomAD_exome_SAS_Ref_freq	gnomAD_exome_SAS_Alt_freq	gnomAD_exome_OTH_Ref_freq	gnomAD_exome_OTH_Alt_freq
0.9999	1.00E−04	1	0	1	0	0.9997	3.00E-04	1	0
0.9999	1.00E−04	1	0	1	0	1	0	1	0
0.9987	0.0013	1	0	1	0	1	0	1	0

Func.knownGene	Gene.knownGene	ExonicFunc.knownGene	AAChange.knownGene
exonic	SMAD4	nonsynonymous SNV	SMAD4:uc002lfb.4:exon4:c.A482G:p.N161S,SMAD4:uc010xdp.2:exon8:c.A947G:p.N316S
exonic	BRCA2	stopgain	BRCA2:uc001uub.1:exon11:c.C3109T:p.Q1037X
exonic	BRCA2	nonsynonymous SNV	BRCA2:uc001uub.1:exon11:c.A4599C:p.K1533N

Downloadable.csv tables as outputs of the TransAT package: (a) Function: convert_transcriptID(), and (b) Function: pop_freq()

### Command 1

convert_transcriptID(userdat,db,biomart_ens = "ensembl",dataset_ens = "hsapiens_gene_ensembl", getBM_attributes_ens = c("refseq_mrna", "ensembl_transcript_id", "hgnc_symbol")).

TransAT can further provide variants for transcript IDs of model organisms such as zebrafish (*Danio rerio*) and mouse (*Mus musculus*). To obtain variant information from model organisms, users are required to additionally specify *@param dat_ens* = *"drerio_gene_ensembl"* for zebrafish or *@param dat_ens* = *"mmusculus_gene_ensembl" for mouse,* the default being “hsapiens_gene_ensembl".

#### Function pop_freq

To further prioritize queried variants and characterize their functional consequences, variant annotations and gene-based annotations, along with allele frequencies from different global normal populations, can be retrieved using the second TransAT function: “pop_freq”. Such annotation assists in shedding light on variants by identifying overlapped regions of interest. This function can be used directly (without going through the conversion step), depending on the user’s preference. For the latter, i.e., to obtain the annotation for a list of pre-selected variants, the user is required to import a .csv file including information on genomic positions and reference and alternate alleles (Table 1b). Users can choose 1000 Genomes [18], TWB [21], and/or gnomAD [19, 20] using simple specifications, such as “db_1000Genomes_5pop_freq", "db_TWB_GWG_freq" (whole genome array), "db_TWB_NGS_freq" (whole genome NGS), "db_gnomAD_exome_freq" (whole exome), or "db_gnomAD_genome_freq" (whole genome), respectively, the default being db_gnomAD_exome_freq. Once the command line is executed, the user will be provided with the reference allele and alternate allele frequencies for each variant from their population of interest (Table 2b), along with distribution plots of MAFs across all subpopulations, for the queried variant(s) (Additional File 4: Figure S1). TransAT connects and retrieves population data from MySQL (https://dev.mysql.com/doc/refman/8.0/en/creating-database.html) through the functions dbConnect, and dbGetQuery. TransAT also provides detailed functional gene annotations, which are imported from ANNOVAR. Command 2 shows the command line required to obtain the desired annotations using the pop_freq function.

### Command 2

pop_freq(userdata, pop = "db_gnomAD_exome_freq").

## Results

### Program installation

TransAT is available as an open-source package within the R system and is specifically designed for transcript conversion and annotation. The package is publicly available from the Comprehensive R Archive Network at http://CRAN.R-project.org/. TransAT can be downloaded and installed automatically using devtools::install_github("ShihChingYu/TransAT", force=T) on the R execution page. Thereafter, all functions can be executed by loading the package into R with library(TransAT).

### Example: Pancreatic ductal adenocarcinoma patients

To illustrate how TransAT works, it was used on an example set of transcript IDs from pancreatic ductal adenocarcinoma (PDAC) patients reported in prior studies [23, 24]. PDAC is invariably fatal, with a low 5-year survival rate. Frontline chemotherapy and immunotherapy is ineffective in the majority of patients. Targeted therapies could serve as an alternate treatment strategy to enhance the clinical response rate. PDAC is either sporadic or hereditary [25]. The sporadic type is a combined effect of somatic genomic, genetic, and epigenetic alterations with environmental factors, and the hereditary type, constituting about 5%–10% of all PDAC patients, is caused by germline gene mutations [26]. Therefore, digging into the underlying genetics will lead to identification of the risk factors for PDAC. Once the candidate transcripts are obtained from patient samples, one of the potential ways to proceed with analysis is to (i) validate intronic variants with respect to the respective reference sequences, by mapping the transcripts to the genome, and (ii) obtain the allele frequency of the variants and the corresponding gene mutation information to proceed with downstream analysis, such as association tests or survival analysis. TransAT makes this easy for users, and the workflow can be executed in 2 separate parts (transcript conversion and mapping, and variant annotation) based on the user’s requirements.

### Conversion of transcripts to variants

An input (.csv) file with two columns, column 1 with the RefSeq ID (e.g., NM_005359) or UCSC ID (e.g., uc010xdp.2) and column 2 with nucleotide changes along with the CDS position (e.g., A947G, where A->G is the nucleotide change and 947 is the CDS position), is imported. Each row of the input.csv/.BED file should depict each patient transcript (Table 1a, b). The function “convert_transcriptID” then needs to be executed in R (Command 1) with either default options or user-chosen options. To illustrate the mapping of a transcript to genomic locations in TransAT, we selected transcripts from 3 PDAC patients (Table 1a, b) as an example. Table 2a displays the output file providing the users with transcript specifics and the corresponding variant information, along with its genomic positions and the gene name. The variant specifics are necessary, as they allow the user to obtain further annotation information and identify pathogenic variants. The results show that the transcripts from the 3 PDAC patients are mapped to a variant from *SMAD4* (transcript: NM_005359) [23] and two variants from *BRCA2* (transcripts: NM_000059) [24]. Transcript NM_000059 is found in both patients and both belong to gene *BRCA2*; however, due to splicing, they are mapped to different genomic positions, thus leading to two different variants. Accumulated genetic alterations play important roles in the tumorigenesis of PDAC, and several somatic mutations in *SMAD4* and *BRCA2* have been found to play important roles in the tumorigenesis of PDAC [24, 27]. Moreover, studies have noted that dysregulation of alternative splicing that generates functionally diverse protein isoforms from a single transcript, is fundamental to cancer, and is the source of novel therapeutic targets [28, 29]. Overall, mapping transcripts to variants that belong to candidate genes is necessary for downstream analysis of clinical outcomes.

### Allele frequency from global populations

Once the genomic positions are obtained, the users can opt to obtain allele frequencies of the respective variants from various global populations using Command 2. Table 2b displays the allele frequencies for the 3 variants (one from *SMAD4* and two from *BRCA2*) from gnomAD subpopulations (from whole exomes) such as American (AMR), African (AFR), and Ashkenazi Jewish (ASJ) ethnic populations. Each row displays information for one variant. The frequency of an allele in the population is a fundamental quantity that is the basis of ethnicity-specific medical genetics studies [30, 31]. Additional File 4: Figure S1, displays distribution of MAFs of the 3 variants, across all sub-populations from gnomAD exome database. Population differences in allele frequencies reveal how SNPs correlate with disease pathogenicity, and population-specific case–control association studies can quantify the difference in allele frequency between affected (cases) and unaffected individuals (controls). Identifying population-specific variants for PDAC patients could eventually help in the development of targeted therapies.

Furthermore, the gene-based annotations (imported from ANNOVAR) [32] are provided to the user for relevant functional information. Table 2b shows all three variants to be exonic (Func.knownGene), with corresponding gene names (Gene.knownGene) and exonic functions (ExonicFunc.knownGene). Variant rs377119288 from *SMAD4* and variant rs80358694 from *BRCA2* are reported to be non-synonymous; variant rs80358557 from *BRCA2* is shown as having stopgain exonic function. The final column displays the amino acid changes (AAChange.knownGene) that may be caused by the mutation. For interpretation of genetic variants with no pre-reported clinical significance, conclusive evidence for pathogenicity of genetic variants is required [10, 33]. Gene annotation information therefore answers questions related to gene functions and their corresponding functional consequences (“functional genomics”). Such information can lead to effective genetic screening, thus providing physicians with ideas for functional tests that can be performed.

## Discussion

A primary hurdle of genome and exome sequencing studies is that the current rate of identification of genetic variation exceeds our ability to interpret its functional consequences. One of several goals of sequencing studies is to identify variants, in a patient under treatment, that are already known to be associated with a disease of interest. This provides guidance for targeted therapy. For other cases, diagnosis is either inconclusive or is based on computational pathogenicity predictions [34]. Therefore, knowledge about added factors such as the type of mutation, change of amino acids, and predicted pathogenicity needs to be acquired and utilized [13]. An existing R package, GenomicFeatures [35], allows users to convert transcriptomic coordinates into genomic coordinates through its function "mapToTranscripts”; however, it fails to provide any annotation information (exon ID or variant IDs), allele frequencies, or gene annotations. Moreover, the input data for the “mapToTranscripts” function needs to be preprocessed by users using the IRanges function, before the conversion can be executed. TransAT, on the other hand, doesn’t require any preprocessing, is extremely convenient, and requires users to only unpack the installed library and execute its functions to obtain all relevant annotation information.

The standards and guidelines for interpretation of genetic variants, proposed by the American College of Medical Genetics and Genomics and the Association for Molecular Pathology [13], lays out several benchmarks that indicate pathogenicity for undiscovered genetic variants. They are as follows: (1) the variant frequency in diseased individuals is significantly higher than in healthy controls, (2) an amino acid change in a patients, whose position concurs with an existing putative variant, (3) a non-functional variant is identified in a gene that coincides with an already putative loss-of-function, (4) a de novo variant with a recognized paternity and maternity, and (5) a deleterious effect identified by already popular functional assays and studies [13]. TransAT comes in handy for establishing the above criteria for a new variant and offers users extensive flexibility to pick and choose annotation types based on their requirements.

## Conclusion

The R package TransAT is the first of its kind. It allows users to provide individual transcript IDs and using two simple functions, provides users with the corresponding variant IDs, genomic locations, allele frequencies from different global populations, and gene annotations. Extremely convenient to use, it has long-term potential for facilitating interpretations of pathogenicity that would benefit bioinformaticians and clinicians alike by providing the annotations of amino acid changes identified from patients, thus revealing genetic differences in populations with distinct genetic backgrounds. We believe it is an important tool that can significantly contribute to the field of biomedical research.

## Supplementary Information

**Additional file 1: ** TransAT: Source Code.

**Additional file 2: Table S1**. Example of BED format. The BED input format for the TransAT package for (a) Function: convert_transcriptID(), which converts transcripts to variants and maps them to genomic positions, and (b) Function: pop_freq(), which provides allele frequencies from global populations and gene-based annotations.

**Additional file 3: Table S2**. Complete list of options from users to choose from, for obtaining variant annotation.

**Additional file 4: Figure S1**. The minor allele frequency (MAF) distribution across sub-populations from gnomAD exome database, for variants corresponding to user queried transcript IDs. (a) Bar plot for transcript ID: NM_005359, and corresponding variant with genomic position 18:48586278A>G. (b) Bar plot for transcript ID: NM_000059, and corresponding variant with genomic position 13:32911601C>T. (c) Bar plot for transcript ID: NM_000059, and corresponding variant with genomic position 13:32913091A>C.

## Data Availability

No raw data were used in this study. All data generated or analyzed during this study are included in this published article and its supplementary information files.

## Abbreviations

TransAT: Transcript annotation tool
SNP: Single nucleotide polymorphism
RefSeq: Reference sequence database
PDAC: Pancreatic ductal adenocarcinoma
AMR: American
AFR: African
ASJ: Ashkenazi Jewish
TWB: Taiwan Biobank
gnomAD: The Genome Aggregation Database

## Declarations

### Availability and requirements

Project name: TransAT. Project home page: https://github.com/ShihChingYu/TransAT. Operating system(s): Platform independent. Programming language: R. Other requirements: R version 4.0.4 or higher. License: GPL-2. Any restrictions to use by non-academics: None.
