# Supplementary material for: Transcript annotation tool (TransAT): an R package for retrieving annotations for transcript-specific genetic variants
Source: BMC Bioinformatics. 2021 Jun 28;22:350. doi: 10.1186/s12859-021-04243-z (PMC8240296; doi:10.1186/s12859-021-04243-z)
Supplement: Supplementary file 1 — Additional file 1: TransAT: Source Code. [file 12859_2021_4243_MOESM1_ESM.docx]

**Supplementary Materials**

**TransAT : Source Code**

**Project name:**TransAT
**Project home page:**[https://github.com/ShihChingYu/TransAT](https://github.com/ShihChingYu/MRAT)
**Operating system(s):**Platform independent
**Programming language:**R
**Other requirements:**R version 4.0.4 or higher
**License:** GPL-2
**Any restrictions to use by non-academics:** None

All source Codes are freely available at:

GitHub links:

1. Function ***convert_transcriptID:*** <https://github.com/ShihChingYu/TransAT/blob/master/R/convert_transcriptID.R>
2. Function ***pop_freq:***

<https://github.com/ShihChingYu/TransAT/blob/master/R/pop_freq.R>

NTU Space link

<https://www.space.ntu.edu.tw/navigate/s/423B06EABCC94300B92179988E36A8DAQQY>
