# Supplementary material for: Transcript annotation tool (TransAT): an R package for retrieving annotations for transcript-specific genetic variants
Source: BMC Bioinformatics. 2021 Jun 28;22:350. doi: 10.1186/s12859-021-04243-z (PMC8240296; doi:10.1186/s12859-021-04243-z)
Supplement: Supplementary file 2 — Additional file 2: Table S1. Example of BED format. The BED input format for the TransAT package for (a) Function: convert_transcriptID(), which converts transcripts to variants and maps them to genomic positions, and (b) Function: pop_freq(), which provides allele frequencies from global populations and gene-based annotations. [file 12859_2021_4243_MOESM2_ESM.docx]

Table S1. Example of BED format

| **Transcript version** | **CDS position** | **Ref** | **Alt** |
| --- | --- | --- | --- |
| NM_000075 | 763 | C | T |
| NM_001211 | 874 | G | A |
| NM_001113378 | 3236 | C | T |
| NM_001166110 | 151 | C | T |

The BED input format for the TransAT package for (a) Function: convert_transcriptID(), which converts transcripts to variants and maps them to genomic positions, and (b) Function: pop_freq(), which provides allele frequencies from global populations and gene-based annotations.
