# Supplementary material for: Transcript annotation tool (TransAT): an R package for retrieving annotations for transcript-specific genetic variants
Source: BMC Bioinformatics. 2021 Jun 28;22:350. doi: 10.1186/s12859-021-04243-z (PMC8240296; doi:10.1186/s12859-021-04243-z)
Supplement: Supplementary file 4 — Additional file 4: Figure S1. The minor allele frequency (MAF) distribution across sub-populations from gnomAD exome database, for variants corresponding to user queried transcript IDs. (a) Bar plot for transcript ID: NM_005359, and corresponding variant with genomic position 18:48586278A>G. (b) Bar plot for transcript ID: NM_000059, and corresponding variant with genomic position 13:32911601C>T. (c) Bar plot for transcript ID: NM_000059, and corresponding variant with genomic position 13:32913091A>C. [file 12859_2021_4243_MOESM4_ESM.docx]

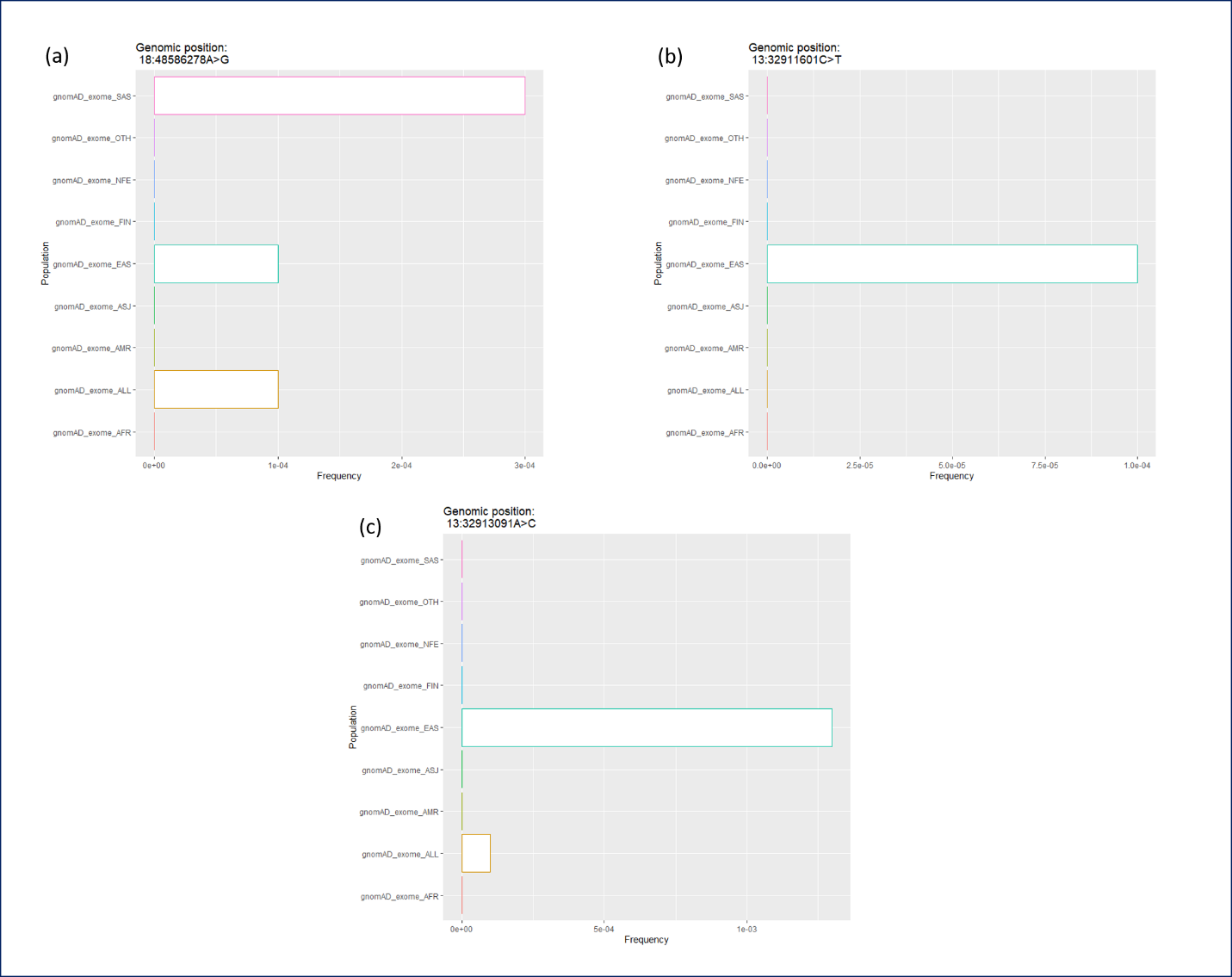


Figure S1. The minor allele frequency (MAF) distribution across sub-populations from gnomAD exome database, for variants corresponding to user queried transcript IDs. (a) Bar plot for transcript ID: NM_005359, and corresponding variant with genomic position 18:48586278A>G. (b) Bar plot for transcript ID: NM_000059, and corresponding variant with genomic position 13:32911601C>T. (c) Bar plot for transcript ID: NM_000059, and corresponding variant with genomic position 13:32913091A>C.
